# Transcriptome Analysis Reveals the Molecular Mechanisms Underlying Adenosine Biosynthesis in Anamorph Strain of Caterpillar Fungus

**DOI:** 10.1155/2019/1864168

**Published:** 2019-12-11

**Authors:** Shan Lin, Zhicheng Zou, Cuibing Zhou, Hancheng Zhang, Zhiming Cai

**Affiliations:** ^1^Department of Central Laboratory, Shenzhen Hospital, Beijing University of Chinese Medicine, Shenzhen University School of Medicine, Shenzhen 518100, Guangdong, China; ^2^Institute of Translational Medicine, Shenzhen Second People's Hospital, First Affiliated Hospital of Shenzhen University, Shenzhen 518035, China; ^3^Zhongshan School of Medicine, Shenzhen Second People's Hospital, Guangzhou 510080, China

## Abstract

Caterpillar fungus is a well-known fungal Chinese medicine. To reveal molecular changes during early and late stages of adenosine biosynthesis, transcriptome analysis was performed with the anamorph strain of caterpillar fungus. A total of 2,764 differentially expressed genes (DEGs) were identified (*p* ≤ 0.05, |log_2_ Ratio| ≥ 1), of which 1,737 were up-regulated and 1,027 were down-regulated. Gene expression profiling on 4–10 d revealed a distinct shift in expression of the purine metabolism pathway. Differential expression of 17 selected DEGs which involved in purine metabolism (map00230) were validated by qPCR, and the expression trends were consistent with the RNA-Seq results. Subsequently, the predicted adenosine biosynthesis pathway combined with qPCR and gene expression data of RNA-Seq indicated that the increased adenosine accumulation is a result of down-regulation of *ndk*, *ADK*, and *APRT* genes combined with up-regulation of *AK* gene. This study will be valuable for understanding the molecular mechanisms of the adenosine biosynthesis in caterpillar fungus.

## 1. Introduction

The Chinese caterpillar fungus, *Ophiocordyceps sinensis* (renamed from* Cordyceps sinensis*) [1], is one of the most outstandingly valued traditional Chinese medicinal fungi [2], and generally found on the Tibetan Plateau with high altitude ranges from 3,600 to 5,400 m [3]. Previous studies have revealed that caterpillar fungus and its anamorph possess a variety of biologically effective ingredients, such as purines, adenosine, polysaccharides, cordycepic acid, ergosterol, with extensive pharmacological effects [4, 5]. Adenosine is considered as an ancient extracellular signalling molecule, which cloud regulate almost all aspects of tissue function, and many previous studies have reported that adenosine plays a positive role of immunity, inflammation, and cancer [6, 7]. Due to the unique medicinal value, the natural sources of shortage and increasing demand, mycelia fermentation of *Cordyceps* fungal species has become a feasible and sustainable mean for producing the medicinal fungus and its bioactive compounds [8, 9]. On the basis of morphological and molecular biology evidences, *Hirsutella sinensis* is currently considered as the only correct anamorph of caterpillar fungus [10, 11]. It was reported that mycelia of *H. sinensis* have similar clinical efficacy and less associated toxicity compared with wild caterpillar fungus, and they are expected to be substitutes of caterpillar fungus [12]. Therefore, with the increasing interests of caterpillar fungus both on mycology and pharmacology, it is becoming more and more urgent to investigate the hereditary information or functional genes of caterpillar fungus by omics sequencing technology.

RNA-Seq is considered as one of the most frequently used methods for transcriptome analysis and gene expression profiling [13], it has many advantages compared with other gene expression profiling technologies, such as allowing a comprehensive assay that does not require probes for targets to be specified in advance [14]. In recent years, the high-throughput RNA-Seq technique has emerged as a useful tool for transcriptome analysis and exploring unknown genes [15]. Gene expression profiles during secondary metabolism are complex. RNA-Seq has been applied to investigate active ingredient accumulation of several traditional Chinese medicine, such as *Ganoderma lucidum* [16], *Paris polyphylla* [17], and *Panax japonicas* [18]. The use of the RNA-Seq technique identified many DEGs that were associated with secondary metabolism. These studies have provided extensive insights into the understanding of significant genetic differences in secondary metabolism. However, the basic molecular mechanism of the active ingredient accumulation in mycelia of fungal medicines, particularly in the different stages of secondary metabolite accumulation, requires further study [19].

Previous studies have been investigated to identify DEGs [20–24]. High-throughput cDNA synthesis and sequencing of poly(A)-enriched RNA were applied to replace microarrays as a quantitative platform for measuring gene expression, and full length cDNA sequencing to 2-channel gene expression microarrays in the context of measuring differential gene expression, as well as sequencing data to a highly replicated microarray experiment profiling two divergent strains of *Saccharomyces cerevisiae* were compared [20]. The hippocampal expression profiles of wild-type mice and mice transgenic for delta-C-doublecortin-like kinase were compared with Solexa/Illumina deep sequencing technology and five different microarray platforms, approximately 2.4 million sequence tags per sample were obtained, and the changes in expression observed by deep sequencing were larger than observed by microarrays or quantitative PCR [21]. In addition, Robinson et al. [22] developed a bioconductor package (edgeR) for examining differential expression of replicated count data, and an over-dispersed Poisson model was conducted to account for both biological and technical variability. These previous studies indicated that it is viable to identify DEGs by different methods with different efficiencies, and developing an efficient method seems meaningful for identifying DEGs.

In the present study, to better understand the molecular factors and their regulatory genes involved in accumulation of active ingredients, the mRNA expression profiles of mycelia between early and late stages of secondary metabolite were compared. We gain insights into the purine accumulation mechanism of caterpillar fungus, particularly the expression of genes in adenosine biosynthesis, as cells transition from early into late stages of secondary metabolite. Physiological observations such as growth and adenosine biosynthesis were linked to transcriptional data obtained first by transcriptomic sequencing, followed by quantitative real-time PCR (qPCR). In addition, GO enrichment as well as KEGG pathway analyses showed that these DEGs were involved in cellular metabolic process, catalytic activity, and biosynthesis of secondary metabolites. These results provide novel insight into understanding the molecular mechanisms of adenosine accumulation and aid in understanding its biosynthesis pathways, and developing future studies on the metabolic regulation of caterpillar fungus.

## 2. Materials and Methods

### 2.1. Strains and Materials

A strain of anamorph of caterpillar fungus was isolated and deposited in our laboratory. Submerged fermentation was performed at 16°C on a rotary shaker at 150 rpm, and mycelia were asexual reproduced and harvested for 10 days. The medium was consisted of tryptone (1%), powdered corn flour (1%), silkworm pupae (1.5%), glucose (1.5%), bran (1.5%), dextrin (0.5%), yeast extract (0.5%), KH_2_PO_4_ (0.02%), and MgSO_4_ (0.01%). The early stage samples of secondary metabolite accumulation were collected for 4-day fermentation, and the late stage samples of secondary metabolite accumulation were collected for 10-day fermentation.

### 2.2. Determination of Mycelia Biomass and Purine Contents

Caterpillar fungus mycelia were cultured at 16°C and collected after cultivation in a shake flask. After washing three times with ultrapure water, the mycelia were dried at 60°C to a constant weight. Assay of purine contents were carried out by HPLC according to a reported procedure with some modifications [25], the column temperature was maintained at 35°C. The standards or samples were separated using a gradient mobile phase consisting of methyl alcohol (A) and ultrapure water (B). The gradient condition is: 0–3 min, 15% A; 3.0–3.5 min, 15–25% A; 3.5–8.55 min, 24% A; 8.5–9.0 min, 24–35% A; 9.0–15.0 min, 35% A; 15.0–16.0 min, 35–85% A; 16.0–22.0 min, 85% A; 22.0–22.5 min, 85–15% A, and 22.5–27.5 min, 15% A. The column was cleaned by 100% methyl alcohol for every 10 runs. The flow rate was set at 1.0 mL/min. The peaks were detected at 260 nm and identified by comparing the retention times with the standard. Standard curves were prepared and the linear regression equation was obtained. The percentage purine extraction yield (mg/g) was calculated as the purine content of extraction divided by dried sample weight.

### 2.3. RNA Isolation, Library Construction, and Sequencing

Total RNA of the anamorph of caterpillar fungus was extracted using TRIZOL and treated with RNase-free DNase I (TaKaRa) according to the manufacturer's protocols (Invitrogen, CA, USA). The mRNA was isolated from total RNA using Promega PolyATtract mRNA Isolation Systems; beads with oligo(dT) were used to isolate poly(A) mRNA. Subsequently, random hexamer-primers were applied to synthesize the first-strand cDNA taking these short fragments as templates, and the second-strand cDNA was synthesized. Sequencing libraries were generated by NEBNext1 Ultra RNA Library Prep Kit for Illumina (NEB, MA, USA). Short fragments were purified with QiaQuick PCR extraction kit and resolved with EB buffer. And then the short fragments were connected with sequencing adapters with respect to the result of agarose gel electrophoresis, and suitable fragments were selected as templates for amplification with PCR. Finally, the library was sequenced using Illumina HiSeq™ 4,000 (Illumina, CA, USA).

### 2.4. Analysis of RNA-Seq Data

The following criteria were applied to remove the sequences: more than 10% unknown nucleotides (N) reads and adapter, and low quality sequence (more than 30% of <Q20 bases). Subsequently, all the clean reads were mapped to the genome by HISAT software. Transcripts assembly was developed by Trinity (https://github.com/trinityrnaseq/trinityrnaseq/wiki) [26]. Then, the assembly results were optimally filtered by TransRate (http://hibberdlab.com/transrate/) [27] and re-evaluated by BUSCO (Benchmarking Universal Single-Copy Orthologs, http://busco.ezlab.org) [28].

The gene expression level was calculated by the normalized number of fragments per kb per million reads (FPKM) method [29]. The DEGs between the early and late stages were identified by RSEM (RNA-Seq by Expectation-Maximization) software by the following filter criteria: *p*-value ≤0.05 as well as absolute value of log_2_ (FPKM_early/FPKM_late)≥1 [30].

### 2.5. Gene Ontology and KEGG Pathway Enrichment Analysis

DEGs were annotated by GO database (http://www.geneontology.org/) using hypergeometric test to examine the biological functions and pathways of these genes. GO functional enrichment analysis provides GO terms which are significantly enriched in DEGs compared to the genome background, showing which DEGs are connected to the wanted biological functions. The analysis firstly maps all DEGs to GO terms in the database (http://www.geneontology.org/), calculating gene numbers for every term, then using ultra-geometric test to find significantly enriched GO terms in DEGs comparing to the genome background [31]. The calculating formula is shown as follows:

(1) $$\begin{array}{c} P\;=\;1-\overset{m-1}{\underset{i\;=\;0}{\sum }}\frac{\left[\underset{\left(n-i\right)}{N-M}\right]\left[\underset{\left(i\right)}{M}\right]}{\left[\underset{\left(n\right)}{N}\right]}, \end{array}$$

where *N* is the number of all genes with GO annotation, *n* is the number of DEGs in *N, M* is the number of all genes that are annotated to the certain GO terms, *m* is the number of DEGs in *M*. The calculated *p*-value went through Bonferroni Correction, taking corrected-*p*-value ≤0.05 as a threshold. GO terms fulfilling this condition were defined as significantly enriched GO terms in DEGs.

KEGG is the major public pathway-related database of biological systems that integrates genomic, chemical, and systemic functional information [32]. KEGG pathway analyses were performed by the KEGG database (http://www.genome.jp/kegg/), the calculating formula is shown as formula (1). Those with a *p* value <0.05 were considered the significant pathways.

### 2.6. qPCR for Verifying DEGs

Verification of RNA-Seq data was performed by qPCR, and 2^−ΔΔCt^ method was conducted to calculate the relative expression levels by comparing the cycle thresholds (CTs) of the target genes with that of the 18S rRNA gene. 17 candidate genes which involved in purine metabolism were selected and validated by qPCR. Differences in relative transcript expression levels were compared at *p* < 0.05 level among different secondary metabolite accumulation periods (early-VS-late) using the Student's *T*-test. Primer pairs of the candidate genes were designed by Primer Express tool (Applied Biosystems, Foster City, USA), and 18S rRNA gene was selected as the internal control (Supplementary [Supplementary-material supplementary-material-1]).

qPCR mixture (10 *μ*L) was prepared and consisted of 1 *μ*L of cDNA from early and late stage samples, respectively, 5 *μ*L of SYBR Green PCR Master Mix (2×) (Promega, Wisconsin, USA), and 0.5 *μ*L (100 *μ*mol/L) of each forward and reverse primer. qPCR analyses were performed three times with independent RNA samples according to the temperature-time profile as follows: denaturation of 95°C for 2 min, 40 cycles of 95°C for 15 sec, 60°C for 1 min.

### 2.7. Statistical Analysis

All experiments in this study were performed in triplicate if not specifically noted. The experimental data were analyzed by the statistical software SPSS (version 9.0, IBM, Chicago). Student's *T*-test and the analysis of variance (ANOVA) test were performed (*p* < 0.05).

## 3. Results and Discussion

### 3.1. Growth Characteristics and Purine Accumulation

Caterpillar fungus grows slowly by artificial culture under suitable conditions, and people attempt to cultivate this fungus for producing its fruiting bodies have frequently failed [33, 34]. To meet the requirement of market, submerged cultivation of caterpillar fungus mycelia provided an environmental-friendly way to resolve this demand [35]. The volatile compound profiles from caterpillar fungus mycelia by submerged cultivation were extracted, and many kinds of active ingredients in the mycelia were observed more abundant than that those in wild caterpillar fungus, indicating submerged cultivation of caterpillar fungus has the trend of gradually replacing the position of caterpillar fungus in market [12].

**Figure 1:**
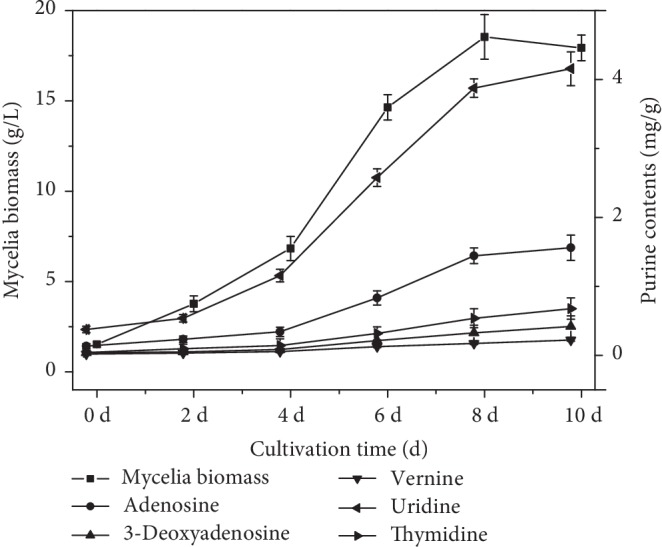
Time course of purine accumulation. Mycelia biomass and purine contents both sharply increased from the early stage (4 d) of secondary metabolite accumulation to and late stage (10 d).

In this study, *H. sinensis* was subjected to growth under optimal culture conditions, and the identified optimal culture conditions were adopted to perform the dynamic profiles of cell growth and purine production. As shown in Figure 1, the mycelia biomass slightly increased until 4 d with 6.83 g/L, and significantly increased to 18.54 g/L at 8 d, then maintained a relatively stable level until 10 d. Furthermore, adenosine and 3-deoxyadenosine production slightly increased until 4 d with 0.342 mg/g and 0.086 mg/g, respectively, and significantly increased to 1.562 mg/g and 0.419 mg/g at 10 d, respectively. Moreover, uridine, vernine, and thymidine production also slightly increased until 4 d and then sharply increased until 10 d. The dynamic profiles of mycelia biomass and purine contents were similar, which all have the same tendency of significant increase after 4 d cultivation (Figure 1). Therefore, the dynamic profiles of cell growth and purine production support our sampling time for early stage of secondary metabolite accumulation at 4 d fermentation, as well as late stage at 10 d fermentation.

A previous study set the sampling time of mycelia at 3 d for growth period of secondary metabolite accumulation and 9 d for stable period, which was earlier than that in our study [31]. However, the present results showed that secondary metabolite increased dramatically from 4 d and maintained a steady level at the end of fermentation until 10 d. Furthermore, a fungal strain UM01 isolated from natural *C. sinensis* was sampled after inoculating for 5 days, while it lacked the investigation on time course of mycelia biomass or production [36]. Therefore, it is not difficult to perceive that 4 d and 10 d were better sampling time points for early and late stages, respectively.

### 3.2. Summary of RNA-Seq Data for Transcriptome Analysis

Secondary metabolite accumulation is crucial for active ingredients and pesticide effects of traditional Chinese medicine. It is well-known that RNA-Seq technique is a powerful approach for transcriptome analysis and exploring unknown genes [37]. Currently, the RNA-Seq technique has been performed in various fungus medicines, including caterpillar fungus, *G. lucidum* [16] and *Cordyceps militaris* [38]. The overriding aim of these studies is to elucidate transcriptome profile changes caused by metabolite accumulation, through comparing results from early and late stages of secondary metabolite accumulation.

In this study, six cDNA libraries from two groups (three from early stage, and three from late stage) were constructed and sequenced. The major characteristics of the sequencing and annotation data are described in Table 1. Subsequently, more than 48 million clean reads for six libraries were obtained after low quality and adaptor sequences were filtered out. Among these clean reads, more than 98.63% and 95.75% had quality scores at the ratio of Q20 and Q30 level, respectively. Moreover, there were 86.48–88.11% of the clean reads mapped onto the reference genome. As shown in Table 2, a total of 68,661 original assembly transcripts and 52,923 optimized assembly transcripts were obtained from the six libraries, and the average transcript length was approximately 1,834.40 for original assembly and 1,279.18 for optimized assembly, respectively. Furthermore, the TransRate score for original assembly and optimized assembly was 0.1929 and 0.40042, respectively. The BUSCO score for original assembly and optimized assembly was 92.7% (15.5%) and 93.4% (15.5%), respectively. Moreover, all transcripts obtained by this RNA-Seq were compared with six databases (NR, swiss-prot, Pfam, COG, GO, and KEGG databases) for functional annotation, and the Venn diagram of functional annotation of transcripts is shown (Supplementary [Supplementary-material supplementary-material-1]). The annotation results showed that number of common comments to the six database was 3,744, and number of unique comments to NR, swiss-prot, Pfam, COG, GO, and KEGG were 3,099, 599, 386, 10, 0, and 137, respectively.

Several studies have reported the transcriptome analysis of entomogenous fungi, and conducted the functional annotation of transcripts. Characterization of the *O. sinensis* transcriptome among three stages of the life cycle was investigated, and a total of 14,922 unigenes were identified and categorized under three gene ontology categories, which were obviously less than those in our study [36]. A previous study detected and analyzed the DEGs of *H. sinensis* growing during different days, while each of the growth and development stages did not be compared [31]. In another study, the transcriptome of the medicinal *O. sinensis* fruiting body was analyzed and a total of 34,289 unigenes were obtained, but the unigenes involved in growth and development stages were not analyzed [39]. In this study, the DEGs from early and late stages were identified and analyzed, which would be useful for the further study of secondary metabolite accumulation.

### 3.3. Expression Analysis and Identification of DEGs

Because of the importance of RNA-Seq, a lot of methods have been conducted to analyze RNA-Seq data for identification of DEGs in recent years, including edger [22], bay_Seq [40], DE_Seq [41], and NBP_Seq [42]. The majority of these methods are based on Poisson or negative binomial distributions when they are dealing with RNA-Seq count data [43]. However, FPKM is the most frequently used measure of mRNA abundance based on RNA-Seq data [44], it is calculated from the number of fragments mapped to a particular gene region with a feature length, which is the number of nucleotides in a capable region of a gene [24].

In this study, a FPKM method for identification of DEGs with RNA-Seq data was developed, and the FPKM of each unigene/transcript for early and late samples wes calculated, as well as the value of log_2_ (late_FPKM/early_FPKM), *p*-value of statistical test and FDR were calculated, if the value of log_2_ (late_FPKM/early_FPKM) > 0, then this gene is up-regulated at late stage, or else it is down. Overall distribution of unigene/transcript expression in each sample is shown (Supplementary [Supplementary-material supplementary-material-1]). The box plot of expression distribution presented that each box graph corresponds to five expression statistics (maximum, upper quartile, median, lower quartile, and minimum). Meanwhile, the violin plot of expression distribution presented that enlarged portion of the image represents the region with the highest concentration of unigene/transcript expression in the sample.

**Figure 2:**
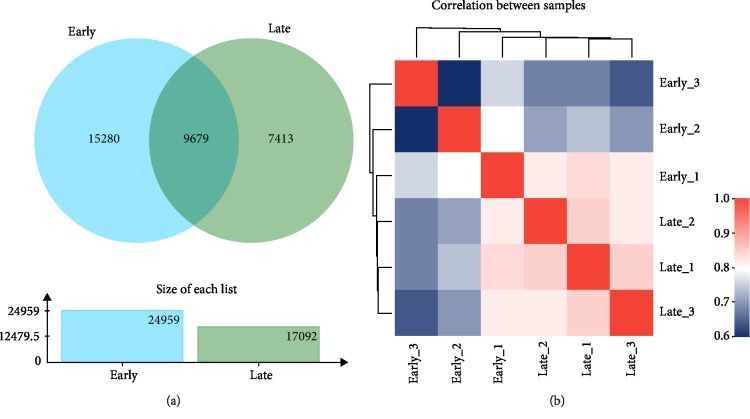
Expression analysis between early and late samples based on the expression matrix. (a) Venn analysis between early and late samples, the intersecting areas of the circles represent the number of unigene/transcript common to each group, inter-sample Venn analysis can obtain co-expression and specific expression genes/transcripts between samples and groups. (b) Inter-sample correlation analysis, different colors represent the size of the correlation coefficient between samples. Correlation analysis helps to understand the correlation between samples, especially among biological duplicates.

**Figure 3:**
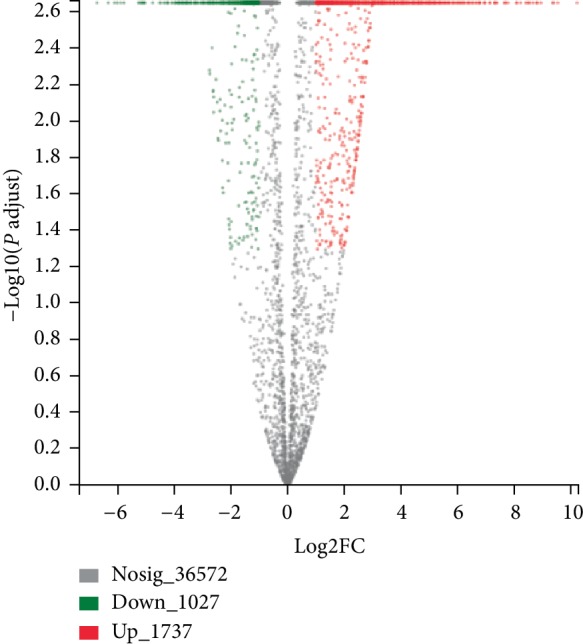
Volcano plot of differentially expressed genes. The abscissa is the multiple change value of gene expression difference between two samples. The ordinate is the statistical test value of the variation of gene amount. The red dots indicate significantly upregulated unigene/transcript, the green dots indicate significantly downregulated unigene/transcript, the black dots indicate no significantly expressed unigene/transcript.

Based on the expression matrix, Venn and correlation between samples were analyzed. As shown in Figure 2(a), there were 9,679 common elements between early and late stages, indicating co-expression and specific expression genes/transcripts between samples could be obtained by inter-sample Venn analysis shown. As shown in Figure 2(b), correlation analysis helps to understand the correlation between samples, especially among biological duplicates. Among the six cDNA libraries, a total of 39,336 genes were detected and the FPKM method was utilized to evaluate the gene expression level (Figure 3). In order to analyze the transcriptome differences between early and late stages of secondary metabolite accumulation, the late stage was compared to the early stage. A total of 2,764 significant DEGs were identified, of which 1,737 genes were up-regulated and 1,027 genes were down-regulated (*p* value ≤0.05 and |log_2_ FC| ≥1).

### 3.4. Gene Ontology Functional Annotation and Enrichment of DEGs

GO is an international standardized gene functional classification system which offers a dynamic-updated controlled vocabulary and a strictly defined concept to comprehensively describe properties of genes and their products in any organism [30]. In order to offer a dynamic-updated controlled vocabulary and a strictly defined concept to comprehensively describe properties of DEGs and their products in any organism, GO functional classification annotation, which includes three ontologies (molecular function, cellular component and biological process) were conducted [45].

**Figure 4:**
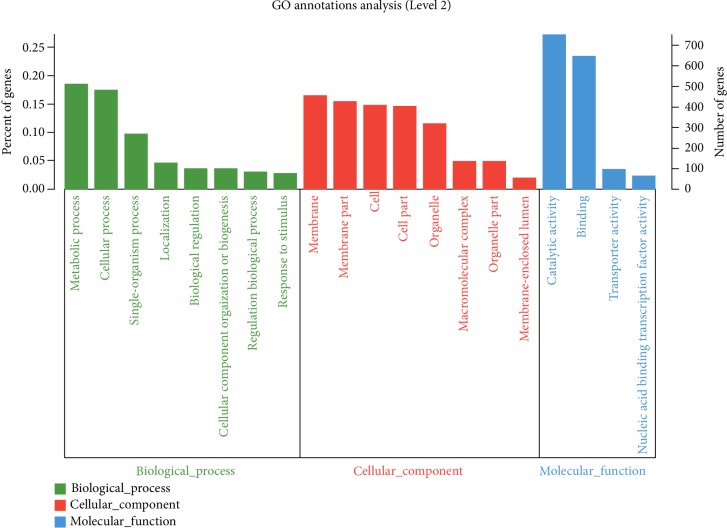
GO classification statistics of DEGs. The abscissa in the figure represents the secondary classification of GO. The vertical axis represents the percentage of the total unigene/transcript contained in this secondary classification.

As shown in Figure 4, in the GO category of biological process, DEGs were involved in metabolic process (513 DEGs), cellular process (485 DEGs), single-organism process (268 DEGs), localization (127 DEGs), biological regulation (99 DEGs), cellular component organization or biogenesis (98 DEGs), regulation of biological process (84 DEGs), and response to stimulus (75 DEGs). Among the DEGs related to the biological process, the most significant term was metabolic process, indicating that metabolic process was extremely active during secondary metabolite accumulation. In the GO category of molecular functions, DEGs were involved in membrane (452 DEGs), membrane part (430 DEGs), cell (408 DEGs), cell part (404 DEGs), organelle (317 DEGs), macromolecular complex (135 DEGs), organelle part (135 DEGs), and membrane-enclosed lumen (55 DEGs). It was indicated that the enriched terms were potentially associated with the secondary metabolite accumulation, and the most significant term located in the membrane played the most important role during secondary metabolite accumulation. In the GO category of molecular functions, DEGs were involved in catalytic activity (754 DEGs), binding (646 DEGs), transporter activity (96 DEGs), and nucleic acid binding transcription factor activity (63 DEGs). The major molecular function category was catalytic activity, indicating that a large number of enzymes were involved in the synthesis of secondary metabolites.

Furthermore, GO functional enrichment of genes/transcripts in gene concentration was analyzed by Fisher's exact test. When the adjusted *p* value (*p* adjust) was <0.05, this GO function was considered to be significantly enriched. As shown in Supplementary [Supplementary-material supplementary-material-1], DNA integration, peptide biosynthetic process, cellular protein metabolic process, and biosynthetic process were significantly enriched. Among them, DNA integration had the maximal rich factor, indicating that DNA integration played an important role in cell growth and secondary metabolite accumulation.

### 3.5. KEGG Pathway Annotation and Enrichment of DEGs

**Figure 5:**
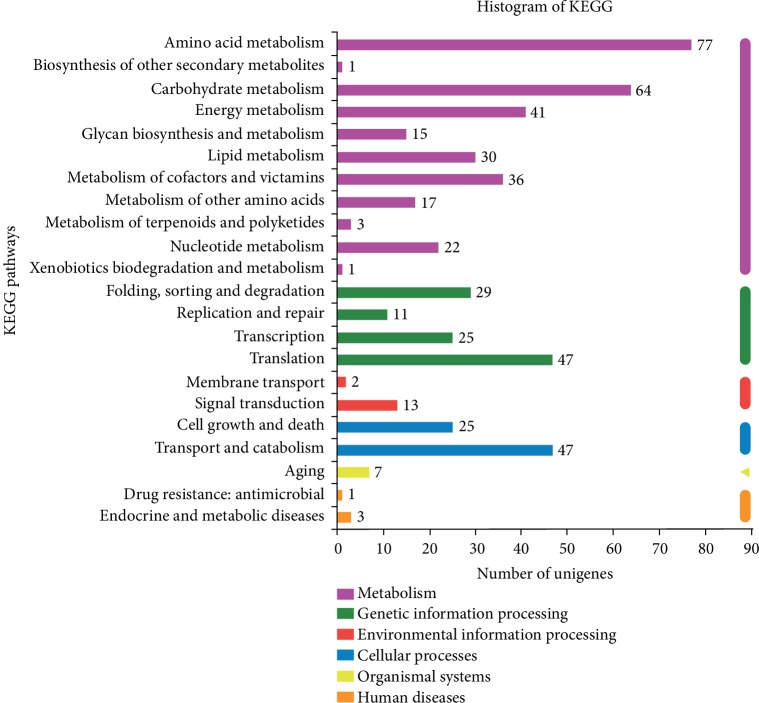
Histogram of KEGG pathway classification statistics. The ordinate is the name of KEGG metabolic pathway, the abscissa is the number of transcript/unigene annotated into the pathway.

Different genes usually cooperate with each other to exercise their biological functions, and pathway-based analysis helps to further understand genes biological functions [46]. KEGG is the major public pathway-related database. Pathway enrichment analysis could significantly enrich metabolic pathways or signal transduction pathways in DEGs compared with the whole genome background [43]. A KEGG pathway analysis was developed to identify the pathways of the DEGs involved in secondary metabolite accumulation. Among the 2,764 late-VS-early DEGs, there were 636 DEGs with pathway annotation, and the results of main pathway enrichment analysis of late-VS-early DEGs are shown in Figure 5. Totally, 636 DEGs were mapped to 104 KEGG pathways, and 22 pathways were significantly enriched (*p* ≤ 0.05). In the significant pathways, several main pathways were represented, including amino acid metabolism, carbohydrate metabolism, energy metabolism, metabolism of cofactors and vitamins, translation, transport, and catabolism.

KEGG pathway enrichment analysis was carried out on genes/transcripts in gene concentration by Fisher's exact test. When the adjusted *p* value (*p* adjust) was <0.05, this KEGG function was considered to be significantly enriched. As shown in Supplementary [Supplementary-material supplementary-material-1], cell cycle-yeast, phenylalanine metabolism, tyrosine metabolism, glycerophospholipid metabolism, pantothenate and CoA biosynthesis, and various types of *N*-glycan biosynthesis were significantly enriched. Among them, cell cycle-yeast had the minimum *p* value, indicating that cell cycle-yeast was most significantly enriched and played the most important role in cell growth and secondary metabolite accumulation.

### 3.6. Verification of DEGs Involved in Purine Metabolism

Differential expression analysis was frequently conducted to screen DEGs, and qPCR was commonly applied in relative expression levels analysis for decades to verify DEGs [47–49]. Gene annotation and differential expression analysis by qPCR identified 464 transcripts that may be involved in catabolism and metabolism of phytohormone, and relative expression levels analysis showed that eleven phytohormone-related genes have different expression patterns in the seed stratification process of *P. polyphylla* [47]. Two proteases which are known to be directly involved in the process of pathogenesis in entomopathogenic fungi *Beauveria bassiana* were identified through a comparative analysis of gene expression patterns and verified them by qPCR [48]. The differential expression of thirteen PHB accumulation related genes was investigated by qPCR, indicating thirteen most up-regulated genes played important roles in PHB metabolism in *Acidiphilium cryptum* [49]. These previous studies suggested that qPCR is a reliable way to verify the DEGs involved in metabolic pathway.

**Figure 6:**
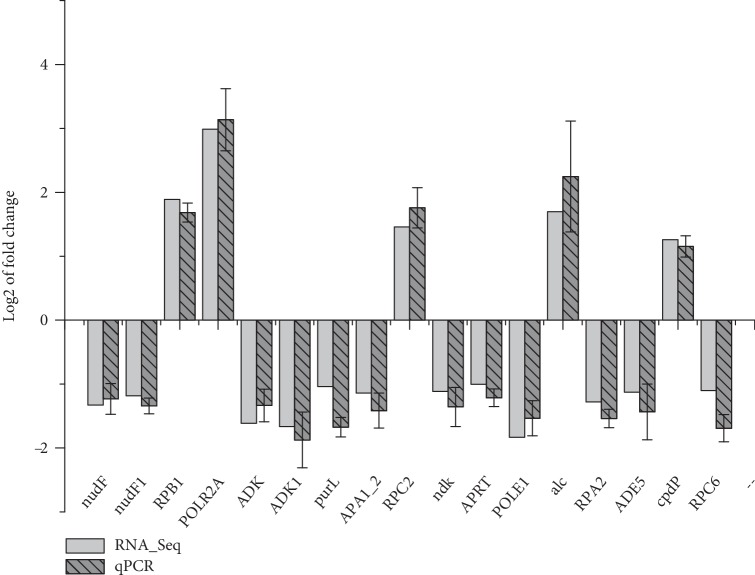
Validation of DEGs which involved in purine metabolism by qPCR. Light gray columns represent the expression level of the DEGs obtained by RNA-Seq, and the dark gray columns represent the qPCR results.

In this study, verification of the selected DEGs was conducted by qPCR, and the results are shown in Figure 6. The 17 candidate genes which involved in purine metabolism (map00230), including ADP-ribose pyrophosphatase (*nudF, nudF1*), RNA polymerase (*RPB1*, *POLR2A*), adenosine kinase (*ADK*, *ADK1*), phosphoribosylformylglycinamidine synthase (*purL*), ATP adenylyltransferase (*APA1_2*), RNA polymerase III subunit RPC2 (*RPC2*), nucleoside-diphosphate kinase (*ndk*), adenine phosphoribosyltransferase (*APRT*), DNA polymerase epsilon subunit 1 (*POLE*), allantoicase (*alc*), RNA polymerase I subunit RPA2 (*RPA2*), phosphoribosylamine–glycine ligase (*ADE5*), 3′,5′-cyclic-nucleotide phosphodiesterase (*cpdP*) and RNA polymerase III subunit RPC6 (*RPC6*), were validated by qPCR. Among them, there were 5 up-regulated DEGs, including *RPB1* (3.70-fold), *POLR2A* (7.94-fold), *RPC2* (2.75-fold), *alc* (3.25-fold) and *cpdp* (2.39-fold), and 12 down-regulated DEGs, including *nudF* (0.40-fold), *nudF* (0.44-fold), *ADK* (0.33-fold), *ADK1* (0.32-fold), *purL* (0.49-fold), *APA1_2* (0.45-fold), *ndk* (0.46-fold), *APRT* (0.50-fold), *POLE1* (0.28-fold), *RPA2* (0.41-fold), *ADE5* (0.46-fold), and* RPC6* (0.47-fold). Although the fold change varied between the two methods, the trends in the expression of the 17 genes were consistent with the RNA-Seq results, suggesting that the RNA-Seq results were reliable.

Indeed, there were few studies that focused on the purine metabolism in the anamorph of caterpillar fungus. The purine metabolic pathway in *C. militaris* was constructed based on the KEGG annotations, and the genes putatively involved in purine metabolism were obtained, while the verification of the putative genes was not conducted [50]. In addition, purine biosynthesis pathway of *H. sinensis* was predicted, which starts from adenosine and ends with urate after 7 steps of catalysis, but the pathway was not systematic and the DEGs involved in purine biosynthesis were not analyzed [31]. In the present study, purine metabolism was more systematically analyzed, and the DEGs involved in purine metabolism were investigated and validated, which could provide useful information for further metabolic regulation.

### 3.7. Construction of Adenosine Metabolic Pathway

**Figure 7:**
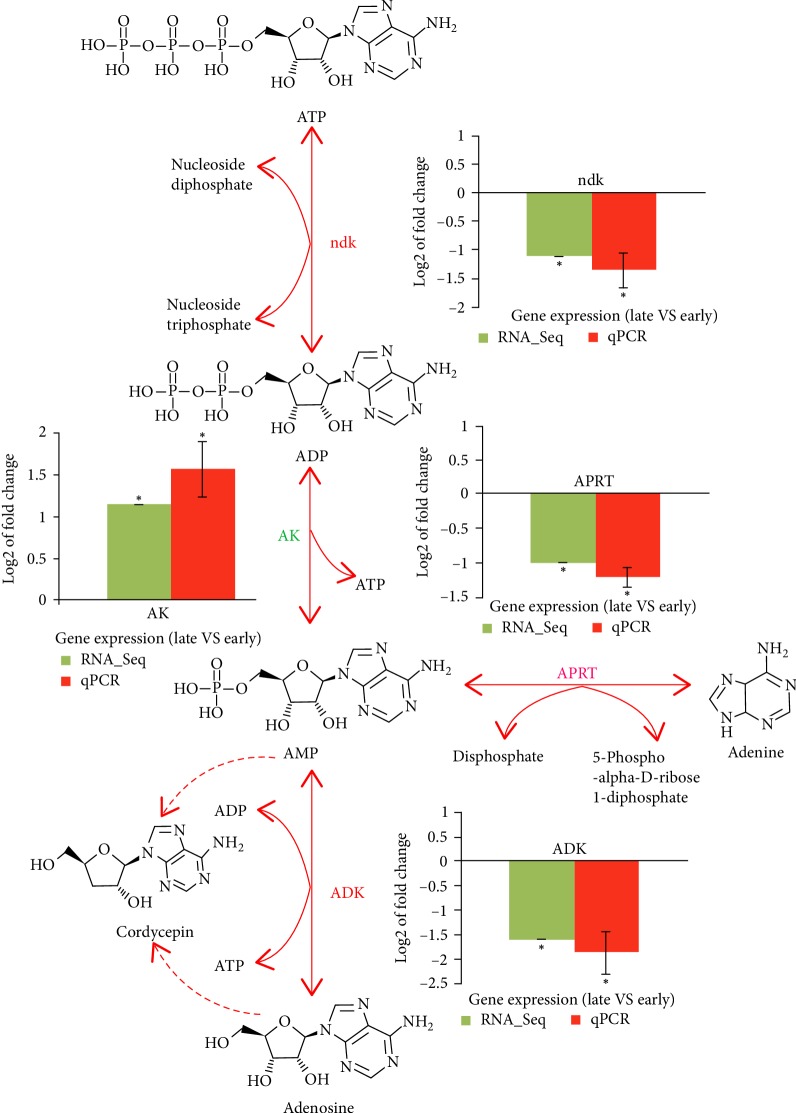
The predicted adenosine biosynthesis pathway and gene expression profiles in caterpillar fungus. Graphs show RNA-Seq and qPCR expression analysis of DEGs at late stage compared with early stage of secondary metabolites biosynthesis. Genes in red font were down-regulated and in green font were up-regulated, with asterisks (∗) indicating significant differences (*p* < 0.05).

Furthermore, based on KEGG purine metabolism (map00230), the predicted adenosine metabolic pathway and gene expression profiles in caterpillar fungus were conducted according to the results of annotation and expression analysis. RNA-Seq and qPCR expression analysis of DEGs at late stage compared with early stage of secondary metabolites biosynthesis were carried out. In adenosine biosynthesis pathway (Figure 7), ATP is converted to ADP by ndk, and ADP is converted to AMP by AK, AMP as an important intermediate in purine metabolism pathway could be converted to adenosine, adenine, and cordycepin, respectively. Among these key genes involved in this pathway, *ndk*, *ADK* and, *APRT* genes were significantly down-regulated, while *AK* gene was significantly up-regulated, which indicated that *ndk*, *ADK*, and *APRT* genes play roles of retro-regulation, while *AK* gene plays the role of positive regulation in the process of adenosine accumulation.

Previously, Zheng et al. constructed metabolic pathways of purine and adenosine based on the KEGG annotation of *C. militaris* to model the cordycepin biosynthesis, and found that adenosine kinase was an important enzyme involved in cordycepin biosynthesis [50]. In addition, a putative biosynthetic pathway was proposed for adenosine in *O. sinensis*, indicating adenosine kinase, adenylate kinase, and 5′-nucleotidase participated in phosphorylation and dephosphorylation in adenosine metabolic pathway [39]. The above previous studies supported the result that *AK* gene plays the role of positive regulation for adenosine biosynthesis, while our results elucidated more detail for the role of other enzymes in the process of adenosine biosynthesis.

## 4. Conclusion

This study provides comprehensive transcriptome data on the early and late stages of secondary metabolite accumulation through RNA-Seq technology. Transcriptome analysis indicated that 2,764 DEGs participated in secondary metabolic synthesis, and 17 of them enhanced purine biosynthesis in caterpillar fungus. The predicted adenosine biosynthesis pathway combined with real-time PCR and RNA-Seq gene expression data indicated that the increased adenosine accumulation is a result of down-regulated of *ndk*, *ADK*, and *APRT* genes combined with up-regulated *AK* gene. This study provides useful information for understanding the molecular mechanisms of accumulation of adenosine, and guiding the metabolic regulation of caterpillar fungus.

## Figures and Tables

**Table 1:** Summary of RNA-Seq data statistics and annotation information.

Samples	Early_1	Early_2	Early_3	Late_1	Late_2	Late_3
Raw reads number	54,028,688	50,541,530	51,518,828	50,358,610	49,729,670	51,270,900
Raw bases	8,158,331,888	7,631,771,030	7,779,343,028	7,604,150,110	7,509,180,170	7,741,905,900
Clean reads number	53,390,400	48,871,682	50,822,684	49,823,872	49,082,804	50,458,096
Clean bases	7,955,067,632	7,261,806,751	7,576,879,005	7,446,100,035	7,324,579,677	7,530,275,238
Clean rate (%)	98.82	96.70	98.65	98.94	98.70	98.41
Q20 (%)	98.72	98.69	98.65	98.72	98.67	98.63
Q30 (%)	95.92	95.88	95.75	95.9	95.8	95.68
GC content (%)	59.75	59.97	60.04	59.79	59.93	59.91
Filtered clean reads	26,695,200	24,435,841	25,411,342	24,911,936	24,541,402	25,229,048
Mapped reads	23,511,904	21,470,372	22,389,649	21,661,602	21,354,130	21,818,903
Mapped ratio	88.08%	87.86%	88.11%	86.95%	87.01%	86.48%

**Table 2:** The evaluation of assembly result.

Source	Original assembly	Optimized assembly
Total transcripts num	68,661	52,923
Total unigenes num	42,289	39,336
Total sequence base	125,951,552	67,697,788
Largest	20,659	20,280
Smallest	201	201
Average length	1,834.40	1,279.18
N50	5,014	3,524
E90N50	3,490	3,914
GC percent	58.44	57.56
Mean mapped reads	1,914.28125609	2,699.98525834
TransRate score	0.1929	0.40042
BUSCO score	92.7% (15.5%)	93.4% (15.5%)

## Data Availability

The data used to support the findings of this study are available from the corresponding author upon request.

## Supplementary Materials

Supplementary Materials Supplementary 1. Table S1: The primers used for real-time PCR. Supplementary 2. Figure S1: Venn diagram of functional annotation of transcripts. Supplementary 3. Figure S2: Overall distribution of unigene/transcript expression in each sample. Supplementary 4. Figure S3: Bubble diagram of GO enrichment analysis. Supplementary 5. Figure S4: Bubble diagram of KEGG enrichment analysis.
