# Supplementary material for: Transcriptome Analysis Reveals the Molecular Mechanisms Underlying Adenosine Biosynthesis in Anamorph Strain of Caterpillar Fungus
Source: Biomed Res Int. 2019 Dec 11;2019:1864168. doi: 10.1155/2019/1864168 (PMC6935459; doi:10.1155/2019/1864168)

Supplementary data for

Transcriptome analysis reveals the molecular mechanisms underlying adenosine biosynthesis in anamorph strain of caterpillar fungus

Authors and their affiliation:

Shan Lin [1,2]*, Zhicheng Zou [1], Cuibing Zhou [1], Hancheng Zhang [1], and Zhiming Cai [1]

[1] Institute of Translational Medicine, Shenzhen Second People's Hospital, First Affiliated Hospital of Shenzhen University, Shenzhen University School of Medicine, Shenzhen 518000, China

[2] Zhongshan School of Medicine, Sun Yat-Sen University, Guangzhou 510080, China

***Corresponding author:** Tel: +86-755-83366388-3230; Fax: +86-755-83366388-3230; E-mail: biotechlin@foxmail.com

Table S1. The primers used for real-time PCR

Genes	Primers	Sequence (5'-3')
18S rRNA	18S-F	GCAGTGGCATCTCTCAGTC
	18S-R	TCATCGATGCCAGAACC
nudF	nudF-F	CCCTCAACGTCACGATTCCA
	nudF-R	AGCACATCCTCCAAACCAGG
nudF1	nudF1-F	GATCCTCAAGGCCCTCATCG
	nudF1-R	AATGTCCTTGTTGGCGTTGC
RPB1	RPB1-F	GAATCTGCTCATGCCTTGCG
	RPB1-R	CGCGCTTCGATTCAGAAAGG
POLR2A	POLR2A -F	CTCCACGTCATTGAAGCCCT
	POLR2A -R	GTAGCCCTGTGCCTCAAACT
ADK	ADK -F	CACCGATAGACCGTTCTCCG
	ADK -R	AGAGTCAGGGGCTCTCGATT
ADK1	ADK1-F	GCGTCGTCACTTGCGTAATC
	ADK1-R	TTCCGGACGTGGACATTGAG
purL	purL -F	CTACTCGCTCGTACCAGCAG
	purL -R	AAGTTCTGCCTACCGGTTGG
APA1_2	APA1_2-F	CTGCAAATTTCCTAGCGGGC
	APA1_2-R	GGCGTCGTAGTTGTTGAGGA
RPC2	RPC2-F	GGTTTGTCAAACTCCACGCC
	RPC2-R	TCCGATTAGAGGGCGATTGC
ndk	ndk-F	CCAGCCGGGTAAAATGGTCT
	ndk-R	CAGCAGGTTGTCCTTTGTGC
APRT	APRT-F	AGGCTCGTAACGTAGATGCG
	APRT-R	GTTGGACCTCTCCCAGTGTG
POLE1	POLE1-F	TTGCCGAGTTTTTCAAGCGG
	POLE1-R	CCCATTTGTCTACCCCACCC
alc	alc-F	GACCTCGAGATAGCCCTTGC
	alc-R	AACACTTGCCGCTTCGTCTA
RPA2	RPA2-F	TCAAACTCGCTCCCACTTCC
	RPA2-R	TATACCCACTCCTTGCCGGA
ADE5	ADE5-F	AGACGGCTCACAACATCCTG
	ADE5-R	CGTGACAGTCTCGGGATACG
cpdP	cpdP-F	GGTGTCTCGTCCAAGAACGT
	cpdP-R	CAAGAAAGCTCGCTCGATGC
RPC6	RPC6-F	GCGACAAACCATGACAACGG
	RPC6-R	CATGCTGGCCTCGGAGAC

Figure S1. Venn diagram of functional annotation of transcripts.

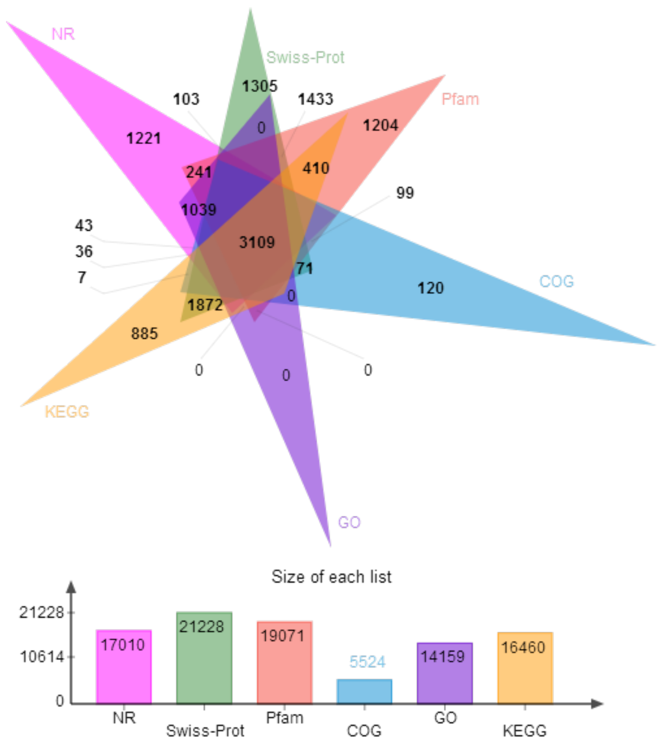

Figure S2. Overall distribution of unigene/transcript expression in each sample. The abscissa is the sample name, and the ordinate is the log value of the expression amount of unigene/transcript of each sample. (A). Box plot of expression distribution, each box graph corresponds to five expression statistics (maximum, upper quartile, median, lower quartile, and minimum). (B). Violin plot of expression distribution, the enlarged portion of the image represents the region with the highest concentration of unigene/transcript expression in the sample.

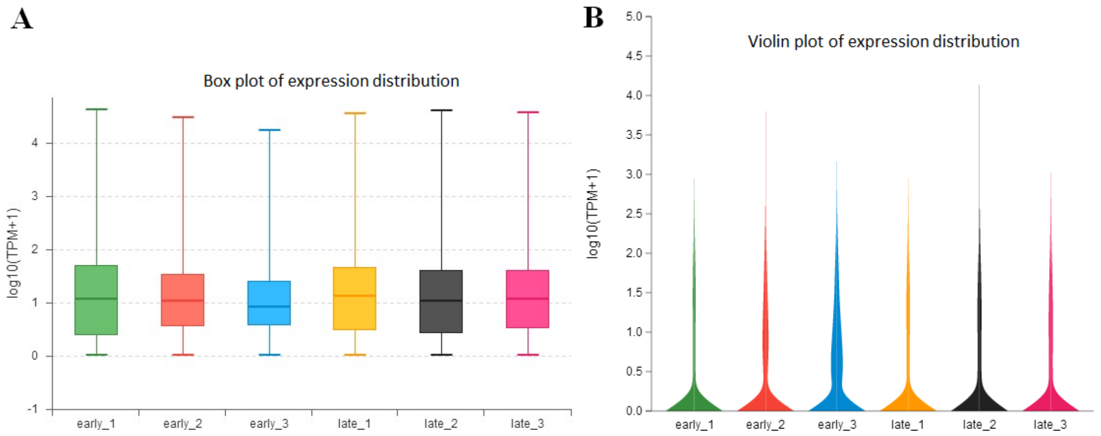

Figure S3. Bubble diagram of GO enrichment analysis. GO enrichment analysis was carried by Goatools, and the GO functions of genes concentrated in this gene were obtained. The vertical axis is GO term, the horizontal axis is the Rich factor.

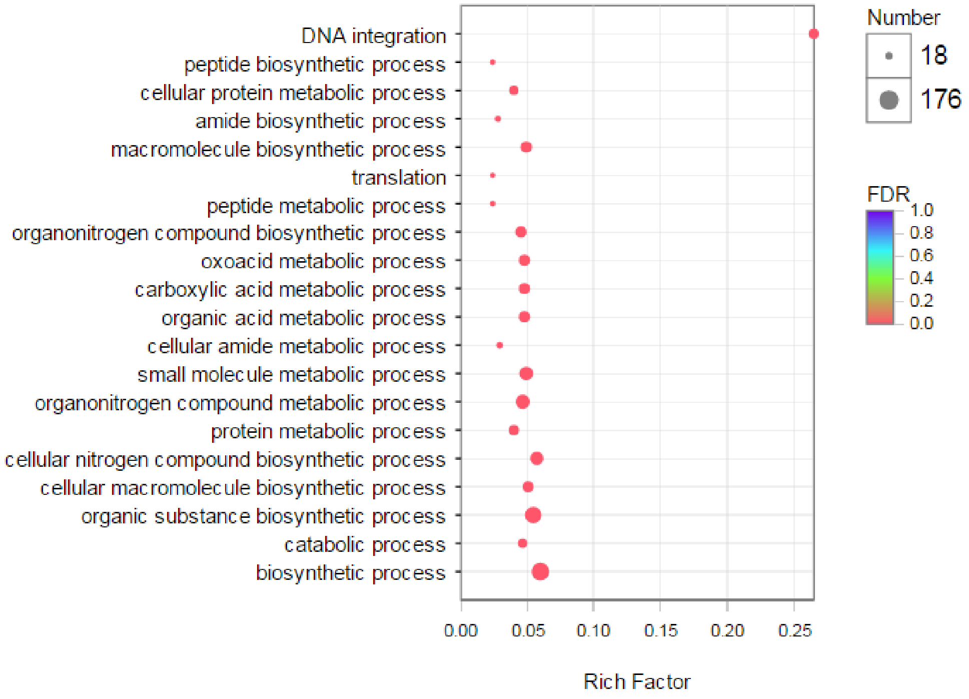

Figure S4. Bubble diagram of KEGG enrichment analysis. KEGG PATHWAY enrichment analysis was carried out to obtain which metabolic pathways the genes in the gene concentration were mainly involved in. Fisher's exact test was used. When the corrected P value (FDR) is < 0.05, this KEGG pathway was considered to be significantly enriched in gene concentration. The vertical axis is pathway term, the horizontal axis is the Rich factor.

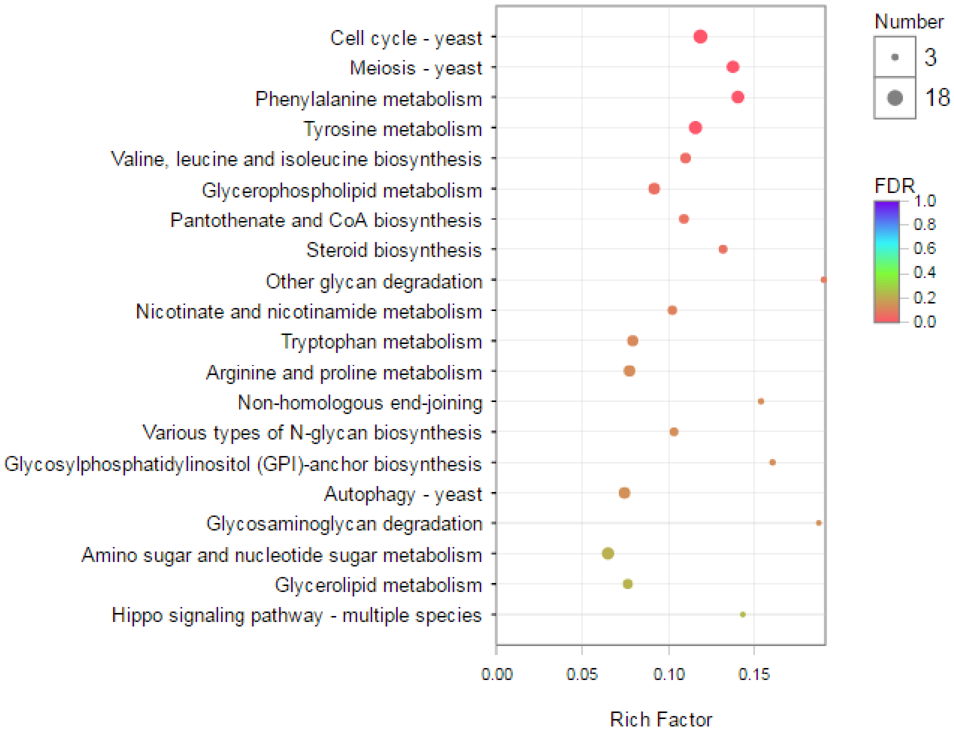

Supplement: Supplementary Materials — Supplementary 1. Table S1: The primers used for real-time PCR. Supplementary 2. Figure S1: Venn diagram of functional annotation of transcripts. Supplementary 3. Figure S2: Overall distribution of unigene/transcript expression in each sample. Supplementary 4. Figure S3: Bubble diagram of GO enrichment analysis. Supplementary 5. Figure S4: Bubble diagram of KEGG enrichment analysis. [file 1864168.f1.pdf]
